# What Shall We Do with the Temporary Teeth

**Published:** 1895-11

**Authors:** Emma Eames Chase


ARTICLE II.

### WHAT SHALL WE DO WITH THE TEMPORARY TEETH.

BY EMMA EAMES CHASE, D. D. S.

Read before the Missouri State Dental Association at Pertle Springs, July 10, 1895.

"What shall we do with the temporary teeth ?" is the question that makes me unhappy oftener than any other that arises in daily practice. I find that each added year of practice brings added confidence in the treatment in almost every case in greater proportion than in these of the temporary teeth, so I take pleasure in presenting the problem to you, since Burke says : "He that borrows the aid of an equal understanding, doubles his own."

In looking over the literature on the subject I find many interesting facts. There is no good book written on the subject, but the dental journals and transactions of the dental societies show that there has been no lack of interest. At the very first meeting of the Illinois State Dental Society, May, 1866, three of the eight topics discussed related to deciduous teeth. The subject was taken up again at their next meeting, and has frequently been the subject of discussion since then. The very best men in our profession have thought the problem worthy of study, and have written such excellent papers on the subject that one feels sure that one can add nothing to what has been said. Yet the remembrance of the worry caused by some of these cases urges me to bring the subject before you again.

There is no longer any question as to the importance of preserving the deciduous teeth in health and comfort for their natural lifetime. And this, not because of any fear of irregularity of the permanent teeth which would result from contraction of the jaw following the premature extraction of these teeth, but because of the important function they must fulfill in the development of the entire body, including the permanent teeth and jaws of the child. This brings us to the greatest cause, and, indeed, the foundation of most of the trouble we have with the temporary teeth—that is, the very general and dense ignorance of the people.

How is it possible that this important fact of the vital importance of these "baby" teeth could wholly escape the notice of the mass of the people, when for the past thirty or forty years, at least, it has been talked about in every dental society and written about in every dental journal in the country? Is it because we have failed in our duty? I am quite sure that each one of us does something in the way of educating our patients every day; dropping a hint here and there, as occasion presents; but is this *all* we can do? One feels like Mrs. Partington with her broom, when with a few words, even vigorously spoken words, one meets the wave of ignorance which sweeps into the office from day to day, knowing, as one does, that we touch but the outer edge of a mighty sea.

How can we teach these people who bring to us the child with the aching tooth, for its first experience in the dental chair? How can we reach them before that painful opportunity arises and teach them the value of these organs and the proper care of them; that cleanliness, proper food and use constitute the ounce of prevention that is worth so much more than the pound of cure? This, gentlemen, is the first question I ask you to consider.

But then, the child with the aching tooth having presented itself, what shall we do for it? One of the best writers on the subject says that "to be able to treat deciduous teeth scientifically requires far more circumspection and reflection than would a similar condition in the permanent organs." I am quite sure we all agree with him, and I am not surprised to find that the "Arthur" method, and more recently the "Stebbins" method, or use of nitrate of silver, have been very popular with a class of dentists as short cuts out of a difficult problem. That the practice advocated by each of these gentlemen is often applicable to the deciduous teeth—that they are even advisable in these teeth, when they would not be allowed in the permanent teeth—we must all acknowledge, but we cannot admit that they are available for such universal application as their authors advise. The dressing off of decay and polishing of the surface—of the incisors—for the child of five or six years, or even earlier if the teeth are of fairly good structure, is often better treatment than filling; but one would prefer not to blacken them with nitrate of silver, unless the child were very young and the tooth-structure so poor that there was no other way to save them. But with the proximal surfaces of the temporary molars the "Arthur" treatment, or any treatment which leaves a space between them, is simply barbarous. If you have ever had such a space in your own mouth, be it ever so V-shaped and self-cleansing, you will agree with me.

It is these proximal cavities in the temporary molars that bring me to my second question: "What do you do with them?" I refer particularly to those shallow cavities which involve the entire proximal surface. If the tooth-structure is poor, and the child young, we may be forced to content ourselves with the simple nitrate of silver treatment, which will arrest decay. But if the child is five or six years old, and the cavity retains the food *debris*, it must be filled. The poor, little, thin walls seem to call for an oxyphosphate, but the difficulty of keeping the cavity and filling dry in order to secure the best results from the material, and the force of mastication which breaks down any contour, soon result in a space which again holds the food *debris*; and the last condition of that tooth is worse than the first. As to amalgam in these cavities: the tooth-substance seems almost always to resent it, and sooner or later manages to get rid of it. Nor do I like the gutta-percha or the Hill's stopping, which many find so excellent. It is very liable to crowd down between the teeth, forcing them apart and infringing on the gum until it becomes a source af discomfort. The combination of amalgam and oxyphosphate makes the most satisfactory filling, in my opinion, but the complication of using two different materials in very shallow cavities, or where one must work very quickly, often makes it impossible to accomplish. I found one gentleman who said he filled these cavities with amalgam in one minute and a half; but I fear that most of us are not so dexterous. In regard to the use of nitrate of silver, many people object so seriously to the blackening of the teeth that they will change their dentist rather than have it used, even where they fully understand that it will arrest decay. And usually the people who object most to the blackened appearance are those whose children's mouth, show the greatest neglect. I have found the nitrate of silver very useful in case of pulp exposure, or very sensitive dentine in teeth that are nearing the end of their usefulness, and of which the root is almost absorbed. To cauterize these cavities and then fill without pressure will usually make the tooth perfectly comfortable and useful till the end of its days.

The question of devitalizing the temporary teeth has been so thoroughly discussed in this society that it need not be repeated now. That it is better not to use arsenic, except in the very mild preparation known as "nerve fibre," and that chromic acid or zinc cholide answer the purpose, are generally acknowledged facts and need no discussion. There is no doubt but that a great deal of unnecessary and harmful treatment is inflicted on those deciduous teeth of which the roots are almost absorbed. The outer walls of the roots have not been absorbed, but the entire center of the root and floor of the pulp-chamber are gone, and it is a mistake too often made to try to destroy the pulp in such a tooth.

In regard to periostitis and abscess, the treatment is identical with that of the permanent teeth. As a rule they yield to treatment more readily than do the permanent teeth, but I think most of us meet with obstinate cases of abscess, where, through some difficulty, we are discouraged by a chronic discharge that will not be conquered. Dr. Harlan writes, in answer to my question as to what he did with these abscessed teeth for children between the ages of seven and ten, that he would "treat and fill, generally, at a single sitting."

Another question of vital importance is this: Is it not our duty as the specialist having charge of the teeth of a child, to insist upon proper hygienic care, not only of the mouth, but of the whole body; and to direct a systematic treatment in those cases ef anemic children whose poor teeth and nervous little bodies are the greatest trials of a dentist's trying day? If our friend the oculist or aurist finds a patient in this condition, he will attempt nothing but palliation until the family physician or some specialist has put the entire system in better order, and we, who have the effects of defective nutrition so plainly set before us, certainly owe this duty to our patients, as well as to ourselves.

My last question is: Do you find that teeth of poor structure are benefited by constitutional treatment or special diet?—*Southern Dental Journal.*

